# Large-scale GWAS meta-analysis of serum antibody levels in healthy individuals reveals distinct genetic architectures

**DOI:** 10.1038/s41435-026-00408-z

**Published:** 2026-08-26

**Authors:** Thomas W. Willis, Effrossyni Gkrania-Klotsas, Nicholas J. Wareham, Eoin F. McKinney, Paul A. Lyons, Kenneth G. C. Smith, Chris Wallace

**Affiliations:** 1 Medical Research Council Biostatistics Unit, University of Cambridge, Cambridge, UK; 2 Cambridge Institute of Therapeutic Immunology and Infectious Disease, University of Cambridge, Cambridge, UK; 3 Medical Research Council Epidemiology Unit, University of Cambridge, Cambridge, UK; 4 Department of Infectious Diseases, Cambridge University Hospital NHS Foundation Trust, Cambridge, UK; 5 Department of Medicine, University of Cambridge, Cambridge, UK; 6 The Walter and Eliza Hall Institute of Medical Research, Parkville, VIC Australia; 7 Department of Medical Biology, University of Melbourne, Parkville, VIC Australia

**Keywords:** Genome-wide association studies, Immunogenetics, Antibodies

## Abstract

Antibodies are the principal effector proteins of humoral immunity. Dysregulated antibody production is a feature of a number of heritable immune-mediated diseases, such as the antibody deficiencies and IgA nephropathy. To characterize the common-variant architecture of serum antibody levels in the general population, we conducted the largest GWAS meta-analyses to date of serum IgA, IgM and IgG, attaining sample sizes of 85,204, 55,368 and 58,777, respectively. We identified 82 novel associations across three isotypes, including 38 novel IgA associations (of a total of 77), 36 novel IgM associations (of a total of 82) and 8 novel IgG associations (of a total of 14). We found that isotype-specific genetic architecture was largely disjoint, with few signals colocalising across isotypes in spite of their overall positive genetic correlation. We identified a large number of colocalisations between antibody phenotypes and immune-mediated diseases and 16 between antibody phenotypes and a lymphocyte count phenotype. We report a greatly expanded catalogue of serum antibody-associated variants and characterise these in terms of their pathway context and relation to immune pathology.

**Figure:**
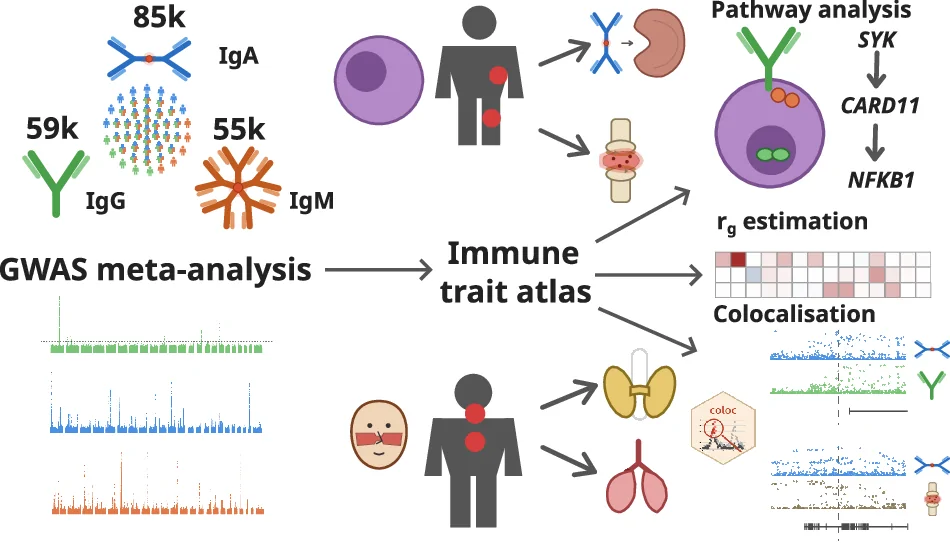

## Introduction

Antibodies are soluble immunoglobulins that form the basis of humoral immunity. They are antigen-binding effector proteins responsible for the neutralisation of pathogens and toxins, opsonisation and the activation of complement. Immunoglobulins also exist as the membrane-bound antigen-binding moiety of cell-surface B-cell receptors (BCRs). In both guises, immunoglobulins are distinguished by the great diversity of antigen-binding affinities they provide to the adaptive immune system, together with T-cell receptors. This diversity depends upon extensive germline polygeny and polymorphism at the *IGH*, *IGK* and *IGL* loci. This germline-encoded variation is supplemented by somatic hypermutation in the germinal centre. In addition to its contribution to antibody repertoire diversity, germline genetic variation also contributes to interindividual variation in the quantity of serum antibody produced [1]. Heritable influences on antibody production have long been studied in the setting of disorders of antibody production: these include antibody deficiencies, such as Bruton’s agammaglobulinaemia [2] and common variable immunodeficiency [3] and hypergammaglobulinaemias, in which there is an excess of antibody production, such as the ‘hyper IgM’ syndromes [4]. Family-based studies in healthy individuals have produced evidence of a substantial heritable component to antibody production [1, 5] and the advent of genome-wide association studies (GWAS) has allowed the discovery of common variants underlying this heritability [6–9].

Antibody dysfunction and the dysregulation of antibody production feature prominently in the pathogenesis of certain diseases such as IgA nephropathy, IgA vasculitis and the aforementioned antibody deficiencies. Study of the common-variant architecture of antibody production in healthy individuals can inform understanding of the genetic basis of these diseases [6, 10] as well as variation in the response to vaccination in the population at large. In this work, we investigate this genetic architecture with two new GWAS of serum IgG and IgM and large-scale meta-analyses of IgG, IgM and IgA. We greatly increase the number of common-variant associations and report updated estimates of serum antibody level heritability and the genetic correlation of isotypes with one another and immune-related traits. We find, contrary to our expectation and to positive phenotypic and genetic correlations overall, that the majority of significant signals detected in these large meta-GWAS are isotype-specific.

## Material and methods

### GWAS of serum IgG and IgM in the EPIC Cohort

We performed GWAS of serum IgG and IgM in 7946 and 8008 individuals, respectively. Participants were drawn from the European Prospective Investigation into Cancer (EPIC)-Norfolk Study (https://doi.org/10.22025/2019.10.105.00004). The study was carried out according to the principles of the Declaration of Helsinki, participants provided informed consent and the study was approved by the Norwich Local Research Ethics Committee. The GWAS were performed using the methodology described previously in our study of serum IgA in EPIC [10]. Briefly, IgG and IgM were quantified using an immunosorbent assay; IgG was measured as the sum of the IgG1, IgG2, IgG3 and IgG4 subclasses. Serum antibody measurements were log-transformed and mean-centred. GWAS was performed for each isotype using an additive model in SNPTEST (v2.5.4-beta3), incorporating age, sex and scores on the first ten genetic principal components.

### Acquisition of public GWAS data sets

We downloaded immunoglobulin GWAS summary statistics [6, 11–14] from the EBI GWAS Catalog where available or from URLs provided in individual publications (Supplementary Table 1). Summary statistics from the Eldjarn study were available both with and without median normalisation; we chose to use median-normalised datasets to match the median normalisation of the Pietzner datasets. Liu et al. published two versions of their IgA meta-analysis, distinguished by the inclusion or exclusion of data from a study of serum IgA in the deCODE cohort [15]. To prevent the inclusion of samples overlapping with those in the Eldjarn study in our own meta-analysis, we used the Liu meta-analysis, excluding the deCODE data. GWAS summary statistics for immune-related traits and diseases [7, 16–27] were downloaded from the URLs in Supplementary Table 2.

### Harmonisation and standardisation of GWAS data sets

Prior to downstream analysis, we processed those GWAS datasets that were not available in a harmonised format from the GWAS Catalog with version 1.1.10 of the EMBL-EBI GWAS summary statistics harmoniser [28]. This process lifted over variant coordinates to the GRCh38 assembly, inferred the strand orientation of palindromic variants and aligned alleles to the Ensembl 95 reference.

The phenotypic values used to produce each GWAS dataset were subject to transformation and standardisation by their respective authors, but the method chosen differed between studies (Supplementary Tables 1 and 3). These differences produced phenotypic values and, therefore, effect estimates and standard errors, on different scales. To correct for this we estimated the standard deviation of the phenotype in each dataset, exploiting the following relationship between phenotypic variance *var(Y)*, genotypic variance *var(X)*, the sample size *n* and the variance of the effect estimate *var(β)*:

$${var}(\beta )\,\cong \,\frac{{var}(Y)}{n\,* \,{var}(X)}$$

where genotypic variance is given by:

$${var}(X)\,=\,2* \,f\,* \,(1-f),$$

with *f* the minor allele frequency estimated using 1 kGP European samples. We regressed n * var(X) on the reciprocal of *var(β)* and obtained an estimate of *var(Y)* as the regression slope. We conducted 10 replicates of this regression in each dataset using 10,000 SNPs randomly selected after LD pruning using *PLINK* version 2.00a5.10 [29, 30] with a 1000 kb window, a step size of one and r^2^ < 0.2. The estimate thus obtained was stable across replicates (Supplementary Table 3). We used the square root of the median of the estimates thus obtained as the estimated standard deviation, which we used in turn as a scaling factor to divide effect estimates and standard errors in each dataset prior to meta-analysis. Our application of this method of standardising summary statistics assumes homogeneity of the underlying phenotype variance across component GWAS; in the absence of phenotype data from these studies, it was not possible to evaluate this assumption.

We did not apply a genomic control correction nor an LDSC intercept correction to the standard errors of the input GWAS nor our final meta-analyses [31].

### GWAS meta-analysis

We performed an outer join between the SNPs of each GWAS for each antibody isotype and then a fixed-effect, inverse variance-weighted meta-analysis.

We designated as candidate ‘lead SNPs’ those variants which had a *p*-value < 5×10^–8^ and the smallest *p*-value in the 1 Mb interval centred upon the variant. We visually inspected the surrounding 4 Mb interval using *locuszoomr* [32] and excluded lead SNPs that appeared to be in LD with a more significantly associated SNP. Estimates of r^2^ reported between pairs of variants in the text were obtained from 1 kGP European samples using the LDlink web application [33].

In order to identify novel associations, we downloaded known associations reported in the EBI GWAS Catalog on February 4th 2025 [28]. We added associations from the GWAS performed by Gudjonsson et al. [34], which we omitted from our meta-analyses due to the possibility of sample overlap with the Icelandic deCODE cohort studied by Eldjarn et al. For IgA, we added associations reported in the second table of the study by Liu et al. after lifting them over to GRCh38 and the associations we reported in our earlier meta-analysis of IgA [10]. Lastly, we added associations present in the studies included in our meta-analyses, except for the studies of IgG and IgM in the EPIC Cohort, which we publish here for the first time. For each of our isotype-specific meta-analyses, we then merged the list of lead SNPs from signals attaining genome-wide significance with our compiled list of known associations using an ‘overlap join’: where no existing association’s reported lead SNP lay within a 500 kb window up- or downstream of the lead SNP in our own meta-analysis, we designated the latter as a novel association.

We downloaded the 2024 edition of the IUIS’ classification table of inborn errors of immunity [35] from the IUIS website (https://iuis.org/committees/iei/) and obtained the HGNC symbol and coordinates of the genes listed in this table from Ensembl using biomaRt [36, 37]. We mapped SNPs to IEI genes which lay within 200 kb.

### Estimation of trait heritability and the genetic correlation

We used the *SumHer* component of *LDAK* version 5.1 to estimate heritability and the genetic correlation between traits [38]. We used the ‘Human Default’ heritability model when estimating the trait heritability and the ‘LDAK-Thin’ heritability model and the ‘SumHer-GC’ model of confounding when estimating the genetic correlation.

We identified highly significant and possibly spurious association signals in the *IGH* and *IGK* loci (Results). We removed these loci (chromosome 14, positions 105,586,437 to 106,879,844 and chromosome 2, positions 88,857,361 to 90,235,368 in the GRCh38.p14 assembly, GenBank Gene IDs 3492 and 50802, respectively) and the *IGL* locus (chromosome 22, positions 22,026,076 to 22,922,913, GenBank Gene ID 3535) for the purposes of heritability and genetic correlation estimation. For the *IGH* and *IGK* loci, it was necessary to exclude a larger region, 103,000,000 to 107,000,000 and 88,700,000 to 90,235,368, respectively, to fully ablate the association signals. We report heritability and genetic correlation estimates with and without inclusion of these loci. We also report heritability estimates with and without inclusion of the major histocompatibility locus (MHC), which we defined liberally as the interval on chromosome 6 between positions 24,000,000 and 45,000,000. The MHC was excluded from datasets for the purpose of genetic correlation estimation. We estimated the phenotypic correlation of isotype-specific serum antibody levels using data from the EPIC Cohort. We used Pearson’s correlation for isotype pairs using both raw and log-transformed data and calculated bootstrap confidence intervals for each estimate using 1000 replicates.

We used SNP genotype data from 535 European-ancestry samples from the Phase 3 release of the 1000 Genomes Project (1 kGP) [39] for out-of-sample estimation of the squared correlation of SNP genotypes (i.e. r^2^) and the calculation of SumHer SNP taggings. All but one GWAS used in our analyses were performed in European-ancestry cohorts (Supplementary Table 1). The exception, the serum IgA meta-analysis of Liu et al., which combined 16 ancestry-defined cohorts spanning European, African, East Asian and admixed ancestries, nonetheless consisted largely of European-ancestry individuals (35,094 of 41,263, ~85%). We therefore identified 1 kGP European-ancestry samples as the most suitable reference panel.

We downloaded data from the high-coverage GRCh38 1 kGP release [40] and processed it using *PLINK* version 2.00a5.10 to meet the following criteria: SNPs only; MAF > 0.005; missingness per SNP < 0.01; missingness per sample < 0.01; exact test of Hardy-Weinberg equilibrium has *p*-value > 1e-50. We enforced alignment of reference and alternative alleles to the GRCh38 reference sequence.

### Annotation of GWAS lead SNPs

We assigned possible causal genes to each lead SNP using the Open Targets Platform [41] and Ensembl version 113 [42]. Genes were assigned to a variant if the variant was missense in the gene or if the variant was in the fine-mapping credible set for any molecular QTL for the gene. For lead variants with no gene thus assigned, we assigned the nearest gene identified using Ensembl annotations. We then performed pathway analysis to identify biological pathways related to these genes as a whole and colocalisation between the different antibody phenotypes, with a lymphocyte count phenotype and with a set of 13 immune-mediated diseases, as detailed below.

### Pathway analysis

To identify biological pathways highlighted by our genetic associations, we first created a test set of genes from the union of possible causal genes across our meta-analyses of IgA, IgG and IgM. We then used the *enrichKegg* function from version 4.14 of the *clusterProfiler* package [43] and the *enrichPathway* function from version 1.50.0 of the *reactomePA* package [44] to test for association with pathways annotated by KEGG and Reactome, respectively.

### Colocalisation

We carried out colocalisation analysis using the *coloc::coloc.abf* function in *coloc* version 5.2.4 [45]. In accordance with package defaults, we defined the prior probability of a random SNP’s being causal for one trait in a pair but not the other as 1 × 10^–4^ and the prior probability of a random SNP’s being jointly causal for both traits as 1 × 10^–5^. We identified candidate serum antibody loci for colocalisation by searching for pairs of lead SNPs for distinct isotypes that lay within 1 Mb of one another. For each pair of lead SNPs thus identified, we included in the analysis all SNPs lying in the interval between the lead SNPs as well as those in the 250 kb flank regions lying up- and downstream of the same interval. For colocalisation of loci for serum antibody and non-serum antibody traits, we searched a 1 Mb interval centred on each serum antibody lead SNP for SNPs reaching genome-wide significance (*p* < 5 × 10^–8^) for the non-antibody trait. Where such SNPs were present, we carried out colocalisation using the SNPs in the 1 Mb interval. We examined regions where the posterior probability of a shared causal variant exceeded 0.5 using the *coloc::plot_extended_dataset* function and designated colocalisations on the basis of the posterior probability of a shared causal variant, the posterior odds of the same hypothesis and the visible concordance of the association signal in each GWAS. The smallest posterior probability of colocalisation for a locus thus identified as the site of a colocalisation was 0.69.

Our serum antibody meta-analyses incorporated multiple studies which differ in the panel of SNPs for which they reported summary statistics. As a consequence, the sample size of each meta-analysis varied across SNPs. Approximate Bayes factors do not account for sample size imbalance of this kind [46], and the resulting miscalibration of fine-mapping [47] may propagate to colocalisation analysis. We conducted a sensitivity analysis, filtering the SNPs included in colocalisation analysis by their sample fraction, i.e. the SNP-specific sample size expressed as a proportion of the largest sample size in the meta-analysis. We retained only those SNPs for which the sample fraction in each meta-analysis differed by no more than 0.1. We found that filtering rarely affected the reported posterior probability of a shared causal variant, with two exceptions (Supplementary Fig. 1); one of these exceptions, the association signal for IgG and IgA at *TNFSF13*, saw the posterior probability of a shared causal variant differ by ~1 between the filtered and unfiltered datasets (Supplementary Figs. 2 and 3). We disregarded this locus from further consideration as the site of a colocalisation.

## Results

**Fig. 1:**
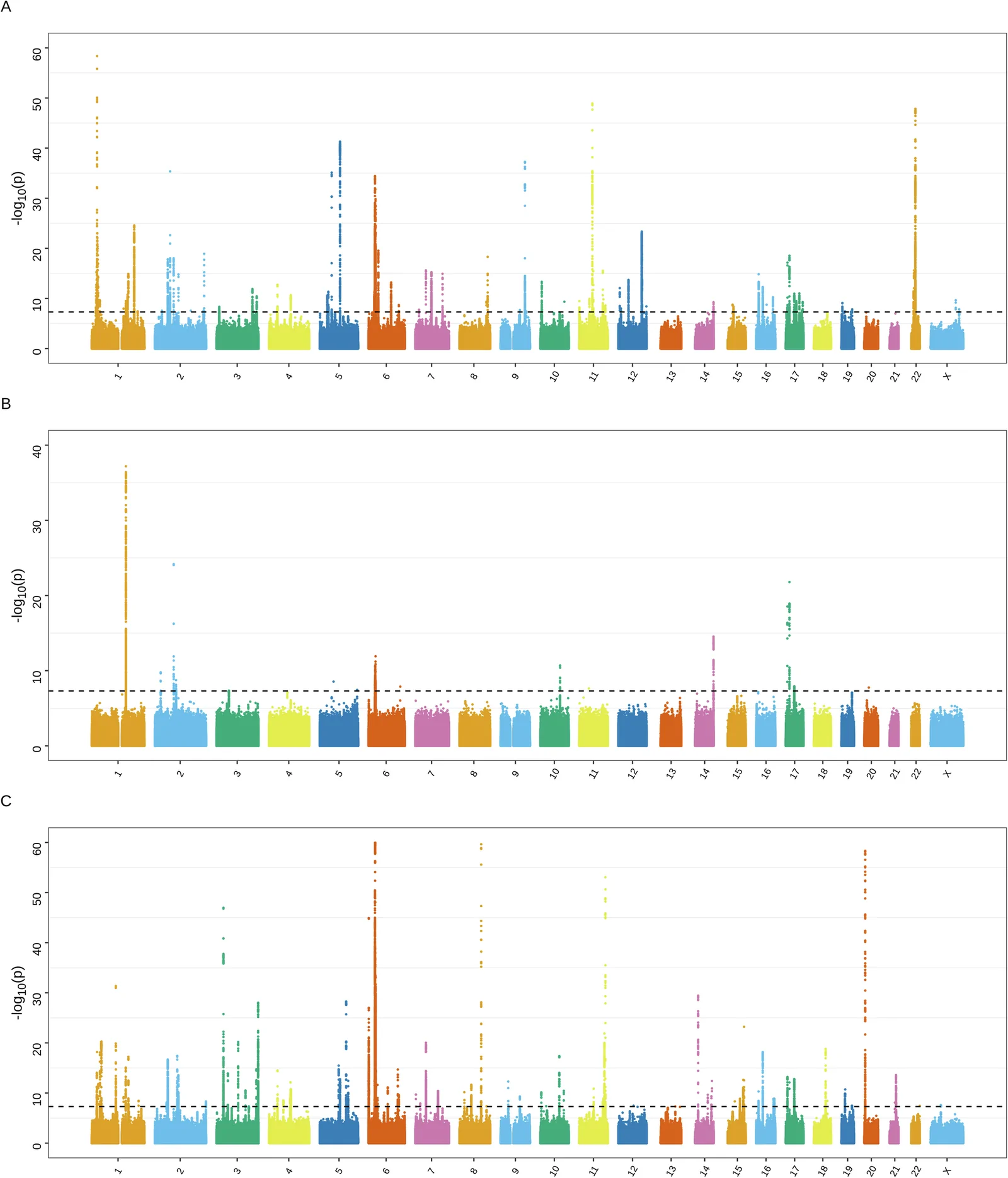
Manhattan plots of isotype-specific GWAS meta-analyses. **A** The serum IgA meta-analysis comprising 85,204 samples. **B** The IgG meta-analysis comprising 58,777 samples. **C** The IgM meta-analysis comprising 55,368 samples. Association signals in the *IGH* region have been omitted owing to the large magnitude of their *p*-values relative to those in other association signals.

We conducted fixed-effect, inverse variance-weighted meta-analyses of IgG, IgM and IgA datasets (Fig. 1 and Supplementary Table 1), attaining sample sizes of 58,777, 55,368 and 85,204, for these isotypes, respectively.

We observed extremely strong associations with variants in the *IGH* and *IGK* loci in multiple studies included in our meta-analyses (Supplementary Table 4). The *IGH*, *IGK* and *IGL* loci contain the genes encoding the heavy and light chain molecules which comprise immunoglobulin tetramers.

Both the antibody-based EPIC study and aptamer-based Pietzner and Eldjarn GWAS of serum IgA and IgG featured genome-wide significant association signals centred upon the constant region of the *IGH* locus. The serum IgA GWAS replicated the known association of the *IGHA1* missense variant, rs117775520, with serum IgA [15], although the direction of effect differed between the EPIC GWAS and the aptamer-based GWAS (Supplementary Fig. 4 and Supplementary Table 4). Variants in the locus were associated with serum IgG in the EPIC, Pietzner and Eldjarn GWAS but the location of the lead SNP differed substantially between EPIC and the other datasets (Supplementary Fig. 5). The Eldjarn GWAS of serum IgM identified a significant but more modest association with rs10873559, near *IGHA1* (Supplementary Fig. 6). rs232230, a missense variant in *IGKC*, was associated with serum IgG in the Eldjarn study (Supplementary Fig. 7). In our meta-analyses, all three isotypes had associations with variants in the *IGH* locus (Supplementary Fig. 8) whereas only IgG and IgA had significant associations in the *IGK* locus (Supplementary Fig. 9).

We could not definitively account for the differing locations and effect directions estimated at *IGH* variants in the serum IgA and IgG GWAS. We hypothesised that coding variation within them (e.g. rs117775520) could modify the immunoglobulin epitopes bound by the capture antibodies and aptamers used in affinity-based quantification and thus induce spurious genotype-dependent variation in antibody measurement. The immunoglobulin allotypes defined by certain coding variants were originally characterised in terms of their differing reactivity to immunologic reagents [48] and recent work has shown IgG1 allotypes do indeed affect detection by commercially available anti-IgG1 antibodies [49]. We therefore took a conservative approach and removed the *IGH*, *IGK* and *IGL* regions from some primary analyses that follow. We report heritability and genetic correlation estimates with the inclusion of these regions for sensitivity purposes.

Outside of the *IGH* and *IGK* loci, we identified 14 associations with serum IgG, of which 8 were novel (Supplementary Table 5), 82 associations with serum IgM (36 novel, Supplementary Table 6) and 77 associations with serum IgA (38 novel, Supplementary Table 7), making for a total of 82 novel associations across all three isotypes (Table 1). We obtained heritability estimates for each isotype using these data (Supplementary Table 8), finding larger estimates for IgA (h^2^ = 0.16) and IgM (h^2^ = 0.21) than for IgG (h^2^ = 0.07). Inclusion of the *IGH*, *IGK* and *IGL* regions increased serum IgG heritability markedly (21.8%) and IgA and IgM heritability modestly (1.5% and 1.6%). We also examined the influence of the major histocompatibility complex (MHC) upon heritability estimates. The MHC features long-range LD patterns and typically evinces strong genetic associations with immune-related traits; SNPs in the MHC featured associations with all three isotypes in our meta-analyses (Fig. 1). Inclusion of the MHC increased estimated IgM heritability the most (22.7%) and had an appreciable effect on IgA (11.0%) and IgG (16.6%), also.

**Table 1:** Lead SNPs from novel genome-wide significant associations in the IgA, IgG and IgM meta-analyses.

Isotype	rsID	Chromosome	Position	Effect allele	Other allele	MAF	Sample size	Gene(s)	Nearest gene	Distance to nearest gene	Missense gene	QTL genes	Beta	Standard error	*p*-value	Q	Degrees of freedom	I2	Q *p*-value
IgA	rs7544568	1	38173536	A	G	0.267222717	85204	POU3F1	POU3F1	126742		POU3F1	-0.035523937	0.005602272	2.28E-10	8.854930087	5	43.53428033	0.114992303
IgA	rs11204701	1	150689703	T	A	0.226003537	85204	MCL1,ENSA,ARNT	GOLPH3L	0		MCL1,ENSA,ARNT	0.033353491	0.005838301	1.11E-08	15.71045858	5	68.17406714	0.007721235
IgA	rs3766151	1	169886435	G	A	0.323642887	85204	SCYL3,FIRRM,KIFAP3	SCYL3	0		SCYL3,FIRRM,KIFAP3	0.034791615	0.005172583	1.74E-11	5.910153273	5	15.3998252	0.315059818
IgA	rs13388394	2	33539109	G	A	0.055073302	84204	RASGRP3	FAM98A	0	RASGRP3		-0.061449273	0.01106481	2.80E-08	4.650623817	4	13.99003322	0.325059046
IgA	rs867529	2	88613755	C	G	0.265603865	85204	EIF2AK3,EIF2AK3-DT,RPIA	EIF2AK3	0	EIF2AK3	EIF2AK3,EIF2AK3-DT,RPIA	0.048301028	0.005464915	9.71E-19	5.239273329	5	4.566918241	0.387382349
IgA	rs62193147	2	230251645	C	T	0.113479849	85204	SP140,SP140L	SP140	0		SP140,SP140L	0.041732538	0.007507485	2.72E-08	5.046055241	5	0.912697908	0.41028546
IgA	rs709167	3	12461456	T	G	0.451285829	77527	MKRN2,PPARG,TSEN2	TSEN2	22964		MKRN2,PPARG,TSEN2	0.030100545	0.00513889	4.70E-09	3.795230056	4	0	0.434427141
IgA	rs2293607	3	169764547	C	T	0.225579602	85204	MYNN,LRRC34,ACTRT3,ENSG00000269984	ACTRT3	2373		MYNN,LRRC34,ACTRT3,ENSG00000269984	0.040890408	0.00576152	1.27E-12	7.170555144	5	30.2703919	0.208261795
IgA	rs55901664	3	177042914	T	A	0.316177076	85204	TBL1XR1-AS1,TBL1XR1	TBL1XR1	0		TBL1XR1-AS1,TBL1XR1	0.02850513	0.005223161	4.83E-08	0.63495225	5	0	0.986347068
IgA	rs17505102	3	189683987	C	G	0.146011956	85204	TP63	TP63	0			0.046354532	0.007009268	3.76E-11	3.487715745	5	0	0.625247202
IgA	rs2101521	4	38809930	A	G	0.25026828	85204	FAM114A1,TLR10,RPL9,TLR1	TLR1	0		FAM114A1,TLR10,RPL9,TLR1	-0.031588069	0.005530622	1.12E-08	11.29275207	5	55.72381323	0.045874786
IgA	rs1585213	4	102523541	T	C	0.406899128	85204	ENSG00000260651,MANBA,UBE2D3,NFKB1	NFKB1	0		ENSG00000260651,MANBA,UBE2D3,NFKB1	-0.03276807	0.004894627	2.16E-11	3.4407743	5	0	0.632367565
IgA	rs6890853	5	35852209	A	G	0.303726572	85204	IL7R	IL7R	485		IL7R	-0.035205366	0.005274629	2.48E-11	5.998225464	5	16.64201304	0.306391627
IgA	rs10440635	5	40490688	A	G	0.402634572	85204	PTGER4	TTC33	21644		PTGER4	0.03405438	0.004924731	4.68E-12	6.766693339	5	26.10866565	0.238577847
IgA	rs6570530	6	142829086	C	T	0.452382313	85204	HIVEP2	HIVEP2	0			-0.029039902	0.004846713	2.08E-09	4.13146777	5	0	0.530647406
IgA	rs79715837	7	17685515	T	C	0.085884291	85204	SNX13	SNX13	105245			-0.05033892	0.008935325	1.76E-08	3.363828302	5	0	0.644084665
IgA	rs1948915	8	127210176	C	T	0.330025834	77527	POU5F1B	POU5F1B	112006			0.033679882	0.005404542	4.61E-10	2.658515294	4	0	0.616493169
IgA	rs4744011	9	90792925	T	C	0.437025088	76527	SYK	SYK	8861		SYK	0.0323204	0.005760143	2.01E-08	3.30499308	3	9.228251696	0.346948305
IgA	rs113495277	9	120913617	G	A	0.031460751	84204	TRAF1	TRAF1	0	TRAF1		-0.09183525	0.01669568	3.79E-08	3.517439873	4	0	0.475231533
IgA	rs1058935	11	308363	C	G	0.475420695	76527	PTDSS2,ENSG00000288681,IFITM1,EPS8L2,PGGHG,IFITM2,IFITM3,IFITM3-AS1	IFITM2	0		PTDSS2,ENSG00000288681,IFITM1,EPS8L2,PGGHG,IFITM2,IFITM3,IFITM3-AS1	-0.039026479	0.006212535	3.34E-10	2.050310764	3	0	0.56203045
IgA	rs1681630	11	47947600	C	T	0.358951741	85204	PTPRJ,F2	PTPRJ	32824		PTPRJ,F2	0.032364269	0.005030911	1.25E-10	0.903712979	5	0	0.969952248
IgA	rs2714073	11	123505241	A	G	0.428127793	85204	GRAMD1B	GRAMD1B	0			-0.029068937	0.004874206	2.46E-09	2.76601476	5	0	0.73600473
IgA	rs1008866	12	12022938	A	G	0.259224188	85204	BCL2L14	BCL2L14	26905			-0.031773828	0.005550018	1.03E-08	2.506566828	5	0	0.775505771
IgA	rs11147102	12	132469613	A	G	0.30053338	77527	ENSG00000256875,FBRSL1	FBRSL1	19937		ENSG00000256875,FBRSL1	-0.033085236	0.005620229	3.94E-09	3.958626155	4	0	0.411634148
IgA	rs539846	15	40105735	T	G	0.473962611	77527	BMF	BMF	0		BMF	-0.031058222	0.005157292	1.72E-09	6.884210703	4	41.89602596	0.142135151
IgA	rs148076268	15	44137670	T	A	0.026263615	84204	MAPDA,PATL2	FRMD5	0		MAPDA,PATL2	-0.090961664	0.015482663	4.23E-09	0.406370542	4	0	0.981952013
IgA	rs12149527	16	79076699	T	C	0.486446925	77527	WWOX	WWOX	0			-0.033643836	0.005137408	5.80E-11	6.595990559	4	39.35709938	0.158841794
IgA	rs2872516	17	39916474	T	C	0.465305709	85204	GSDMA,ZPBP2,ORMDL3,GSDMB,IKZF3	GSDMB	0		GSDMA,ZPBP2,ORMDL3,GSDMB,IKZF3	0.033161686	0.004883678	1.12E-11	5.223467362	5	4.278142209	0.389221237
IgA	rs11655056	17	49286745	T	C	0.432468799	76527	PPP1R9B,ZNF652	ZNF652	2460		PPP1R9B,ZNF652	0.036152266	0.00575482	3.34E-10	1.935100623	3	0	0.585985466
IgA	rs2286564	17	63932631	G	A	0.39789881	85204	FTSJ3,PSMC5,CD79B	CD79B	294		FTSJ3,PSMC5,CD79B	0.033456993	0.004916238	1.01E-11	6.478522494	5	22.82190879	0.262398976
IgA	rs11653912	17	75324509	C	T	0.243816512	76527	GRB2	GRB2	0			-0.041198533	0.007402107	2.61E-08	1.796691684	3	0	0.615655127
IgA	rs3743	17	79935827	A	G	0.345026005	85204	TBC1D16,SLC26A11,CBX2,CBX4,ENSG00000275516	TBC1D16	0		TBC1D16,SLC26A11,CBX2,CBX4,ENSG00000275516	0.031841156	0.00512785	5.32E-10	1.857543621	5	0	0.868484514
IgA	rs7245642	19	4053015	A	G	0.288899628	85204	ZBTB7A	ZBTB7A	0		ZBTB7A	0.03627263	0.005901409	7.92E-10	1.720560443	5	0	0.88629938
IgA	rs754292	19	16589002	T	C	0.472937755	85204	ENSG00000268790		0			-0.026915501	0.00489802	3.90E-08	4.452570754	5	0	0.486254279
IgA	rs8111826	19	45053960	A	G	0.469753835	85204	ENSG00000267114,ZNF234,CLASRP	CLASRP	0		ENSG00000267114,ZNF234,CLASRP	0.026386197	0.004817744	4.33E-08	2.132684601	5	0	0.830497711
IgA	rs3810277	19	50431721	T	G	0.134106884	85204	SPIB	SPIB	406			0.042282753	0.007463711	1.47E-08	5.117849736	5	2.302719736	0.40166774
IgA	rs2256609	22	21570728	G	A	0.200158042	85204	PPIL2,PPM1F,CCDC116,YDJC,UBE2L3,ENSG00000273342,PI4KA	UBE2L3	0		PPIL2,PPM1F,CCDC116,YDJC,UBE2L3,ENSG00000273342,PI4KA	0.042319447	0.006093408	3.78E-12	4.561771951	5	0	0.471661345
IgA	rs3177243	22	23837735	C	G	0.158371144	85204	DERL3,MMP11,SMARCB1	DERL3	0	DERL3	MMP11,DERL3,SMARCB1	0.054594834	0.006661482	2.49E-16	3.52302178	5	0	0.619907039
IgG	rs9864859	3	58286444	C	A	0.249199427	58777	HTD2,ENSG00000273493,FLNB,RPP14,ENSG00000272182,PXK,ABHD6,PDHB	ABHD6	0		HTD2,ENSG00000273493,FLNB,RPP14,ENSG00000272182,PXK,ABHD6,PDHB	0.038388228	0.007019681	4.53E-08	2.139130906	4	0	0.71018712
IgG	rs537870777	5	65146194	T	C	0.00139379	7946	ADAMTS6	ADAMTS6	2543			-1.3812339	0.232487	2.83E-09		1		
IgG	rs7449025	5	176750656	T	A	0.00065536	7946	UNC5A	UNC5A	59862			1.75466722	0.318725151	3.69E-08		1		
IgG	rs759314091	6	149668400	T	C	0.00071105	7946	LATS1	LATS1	0			-1.940938173	0.341609102	1.33E-08		1		
IgG	rs7918084	10	92669710	T	C	0.456952144	58777	HHEX	KIF11	14314		HHEX	0.040827816	0.006087858	1.99E-11	4.702118184	4	14.93195528	0.319249404
IgG	rs184552867	11	45133431	G	A	0.00097673	7946	PRDM11	PRDM11	0			-1.57388056	0.281829268	2.34E-08		1		
IgG	rs17676923	17	39893872	T	C	0.057365726	57777	MIEN1,IKZF3,GSDMB	GSDMB	10722		MIEN1,IKZF3,GSDMB	0.075530136	0.013248055	1.19E-08	2.407382162	3	0	0.492261915
IgG	rs562874417	20	20892169	G	C	0.00113548	7946	RALGAPA2	RALGAPA2	179524			-1.412753783	0.250721663	1.75E-08		1		
IgM	rs12026735	1	30776072	A	G	0.180661834	55368	LAPTM5	LAPTM5	18297		LAPTM5	-0.055995002	0.007919271	1.54E-12	0.592361279	3	0	0.898178836
IgM	rs7536190	1	150426629	A	T	0.112561026	55368	RPRD2	RPRD2	0			-0.058983848	0.009565067	6.98E-10	3.218207948	3	6.780417893	0.359189765
IgM	rs150412347	2	55285800	A	G	0.092990604	55368	RPS27A,CLHC1,CCDC88A	CCDC88A	2041		RPS27A,CLHC1,CCDC88A	-0.060481996	0.010468887	7.59E-09	5.054263698	3	40.64417333	0.16786654
IgM	rs115979345	2	241466693	G	C	0.180832451	54368	FARP2,SEPTIN2,STK25,MTERF4	FARP2	0		FARP2,SEPTIN2,STK25,MTERF4	-0.046932145	0.00801827	4.82E-09	7.762745047	2	74.23591799	0.020622501
IgM	rs28671336	3	136795300	G	C	0.325136945	55368	NCK1,SLC35G2,NCK1-DT	SLC35G2	23346		NCK1,SLC35G2,NCK1-DT	-0.042955161	0.006475	3.27E-11	2.830644888	3	0	0.418480037
IgM	rs1915948	3	152504354	C	T	0.334644183	55368	P2RY1,SUCNR1,ENSG00000243305,MBNL1,RAP2B	MBNL1	38573		P2RY1,SUCNR1,ENSG00000243305,MBNL1,RAP2B	0.035545023	0.006502734	4.60E-08	1.877228027	3	0	0.598275128
IgM	rs1397934	4	40185976	G	A	0.313396175	55368	RHOH	RHOH	5034			-0.051814897	0.006569071	3.08E-15	14.82586034	3	79.7650866	0.001971675
IgM	rs67960624	4	67613670	A	G	0.25714599	55368	YTHDC1,UBA6-DT,UBA6,STAP1,GNRHR	UBA6	0		YTHDC1,UBA6-DT,UBA6,STAP1,GNRHR	-0.040912195	0.006908996	3.19E-09	3.435650187	3	12.6802836	0.329204477
IgM	rs7758383	6	142848586	G	A	0.487925582	55368	HIVEP2	HIVEP2	0			-0.038492649	0.00608555	2.53E-10	2.575898814	3	0	0.461730471
IgM	rs12531349	7	3037424	T	A	0.297879385	55368	CARD11	CARD11	0			-0.042670812	0.006703914	1.95E-10	1.540685717	3	0	0.672912419
IgM	rs73074879	7	23551590	A	G	0.092721121	55368	TRA2A	TRA2A	19548			-0.059664556	0.010475016	1.23E-08	0.694068818	3	0	0.874598129
IgM	rs3823958	7	107614773	C	A	0.457841724	55368	DUS4L,HBP1	BCAP29	0		DUS4L,HBP1	0.040309191	0.006097659	3.83E-11	0.731064686	3	0	0.865874026
IgM	rs2667978	8	55985392	G	A	0.174616079	55368	RPS20,LYN	LYN	0		RPS20,LYN	-0.056217456	0.008010245	2.25E-12	3.116027932	3	3.72358445	0.374079601
IgM	rs12554596	9	37002145	G	A	0.193499275	55368	ENSG00000250850,PAX5	PAX5	0		ENSG00000250850,PAX5	-0.042195342	0.007691863	4.12E-08	6.131896271	3	51.07549333	0.105366574
IgM	rs290243	9	90809055	A	G	0.207959088	55368	SYK	SYK	0		SYK	-0.046500788	0.007476159	4.98E-10	1.323830512	3	0	0.723482214
IgM	rs7918238	10	3876889	A	G	0.458734927	55368	LINC00705	KLF6	91607		LINC00705	-0.039666035	0.006090523	7.38E-11	5.463931763	3	45.09448269	0.140811884
IgM	rs7902629	10	110423817	C	G	0.489588759	54368	DUSP5	DUSP5	74089			0.040776154	0.006158021	3.55E-11	1.832691579	2	0	0.399977978
IgM	rs36027301	11	68041801	T	C	0.057999933	55368	TCIRG1	TCIRG1	0	TCIRG1	TCIRG1	-0.087319699	0.012923577	1.41E-11	0.984597905	3	0	0.804978739
IgM	rs6589225	11	111376136	C	T	0.236523783	55368	POU2AF3,POU2AF1,COLCA1	POU2AF1	0		POU2AF3,POU2AF1,COLCA1	-0.041524783	0.007123124	5.56E-09	3.830571698	3	21.68270857	0.2803501
IgM	rs11215032	11	114519937	G	T	0.112954278	54368	NXPE1,CADM1	NXPE1	0		NXPE1,CADM1	-0.058767743	0.00967189	1.23E-09	4.767167672	2	58.04636762	0.092219485
IgM	rs905688879	12	71786078	G	T	0.00070929	8008	RAB21	RAB21	0			1.741413669	0.31573019	3.48E-08		1		
IgM	rs56356298	13	86109332	T	A	0.00109946	8008	SLITRK6	SLITRK6	302648			1.30651885	0.239572092	4.94E-08		1		
IgM	rs7158350	14	75480111	C	T	0.225805577	55368	YLPM1	JDP2	5999		YLPM1	-0.046407579	0.007313959	2.22E-10	0.578003479	3	0	0.901448988
IgM	rs11627032	14	92637727	C	T	0.259770429	55368	RIN3	RIN3	0		RIN3	0.043070813	0.006909342	4.56E-10	1.532404477	3	0	0.67481184
IgM	rs146125856	15	50492793	C	T	0.09877754	18716	USP8	USP8	0	USP8		0.105916641	0.018078151	4.66E-09	0.781354822	1	0	0.376727107
IgM	rs6495122	15	74833304	C	A	0.420032038	55368	CSK,MPI,SCAMP5,COX5A,FAM219B,RPP25,ULK3	CPLX3	1501		CSK,MPI,SCAMP5,COX5A,FAM219B,RPP25,ULK3	-0.036886304	0.00608997	1.39E-09	1.92122454	3	0	0.588916719
IgM	rs34033173	16	10889076	A	G	0.121373643	55368	CIITA	CIITA	0		CIITA	0.061181188	0.009307947	4.93E-11	7.217718478	3	58.43561911	0.065272771
IgM	rs7194131	16	81831630	A	C	0.113216894	55368	PLCG2	PLCG2	0			-0.057675605	0.009527688	1.42E-09	1.424183031	3	0	0.699876014
IgM	rs305080	16	85908168	T	C	0.35435376	55368	IRF8	IRF8	0		IRF8	0.038513361	0.006357276	1.38E-09	0.969673117	3	0	0.808589604
IgM	rs9907708	17	16989369	T	A	0.368856007	55368	TNFRSF13B	TNFRSF13B	17250			-0.045591626	0.006362164	7.72E-13	10.26911626	3	70.78619111	0.016411763
IgM	rs7236090	18	63279878	C	T	0.473734637	55368	BCL2	BCL2	0			-0.036283403	0.006151723	3.68E-09	6.343175657	3	52.70507767	0.096056739
IgM	rs10419696	19	16309861	G	T	0.309620346	55368	OCEL1	KLF2	14964		OCEL1	-0.042450962	0.006610273	1.35E-10	3.988351892	3	24.78096012	0.262724665
IgM	rs11666267	19	17549491	A	G	0.448256804	55368	COLGALT1,NIBAN3	NIBAN3	0	NIBAN3	NIBAN3,COLGALT1	0.041302352	0.006152356	1.90E-11	5.853616239	3	48.74962967	0.118953416
IgM	rs10412811	19	49381238	A	G	0.250127673	55368	DKKL1,KASH5,ENSG00000197813,ENSG00000268686,SLC6A16,TEAD2,CD37	DKKL1	6121		DKKL1,KASH5,ENSG00000197813,ENSG00000268686,SLC6A16,TEAD2,CD37	-0.038556211	0.00698808	3.44E-08	0.724344387	3	0	0.867463511
IgM	rs192881351	21	29697533	T	C	0.00092795	8008	GRIK1	GRIK1	0			1.651938961	0.286338848	7.97E-09		1		
IgM	rs541130164	22	50704843	T	C	0.0008835	8008	SHANK3	SHANK3	0	SHANK3		1.602511122	0.291310184	3.78E-08		1		

‘MAF’ is the minor allele frequency computed from data in the component GWAS where this information was provided in the original studies. ‘Gene(s)’ gives the gene(s) with the most evidence linking it/them to the association signal; the procedure by which we assigned genes to variants and identified quantitative trait loci (QTL) and missense variants is described in Methods. ‘Missense gene’ reports the gene (if any) in which the lead SNP was identified to be a missense variant. ‘Q’ is Cochran’s Q test statistic for which the degrees of freedom are also given. ‘I2’ is the I^2^ statistic.

All serum immunoglobulin measures were positively correlated on the log scale (Supplementary Table 9). Concordant with this finding were significant positive genetic correlation (r_g_) estimates between serum IgA and IgG and IgG and IgM, when the *IGH*, *IGK* and *IGL* loci were excluded (Table 2 and Supplementary Table 10). These r_g_ estimates were reduced in magnitude and made non-significant with the inclusion of the *IGH*, *IGK* and *IGL* loci. We attribute this to the potential false positive results in the *IGH* locus sufficing to dilute evidence of pleiotropy in the rest of the genome as measured by the genetic correlation.

**Table 2:** Estimates of the genetic correlation between serum antibody isotype levels.

First isotype	Second isotype	Genetic correlation estimate	Standard error	*p*-value
IgA	IgM	–0.029525	0.031657	0.350999094
IgA	IgG	0.208373	0.041774	6.10E-07
IgG	IgM	0.14628	0.045103	0.001181773

*p*-values were calculated from a chi-squared test of non-zero genetic correlation. The MHC and IGH, IGK and IGL loci were excluded from the datasets used to produce these estimates.

In spite of the genetic correlation we observed when omitting these loci, however, we found only one locus at which there was strong evidence of a causal variant shared between IgG and IgA (Supplementary Fig. 10; posterior probability of colocalisation, ‘PPC’, 0.85) and none at which this was true for IgG and IgM (Supplementary Table 11). This may be explained in part by the much smaller number of IgG associations, consistent with its relatively lower heritability estimate (Supplementary Table 8).

In contrast, although IgA and IgM did not show significant genetic correlation overall, six associations with each isotype showed evidence of a causal variant common to both isotypes in colocalisation analysis (Supplementary Table 11, Supplementary Figs. 11–16, minimum PPC = 0.80). For five of these colocalising associations, the direction of effect on serum IgA and IgM was concordant. The exception was on chromosome 4 near *RHOH*, for which association the IgA and IgM lead SNPs had discordant effects on the level of each isotype. *RHOH* encodes the haematopoiesis-specific RhoH GTP-binding protein [50] and mutations in the gene cause an autosomal recessive combined immunodeficiency [51]. RhoH-deficient mice show normal IgM production but impaired production of class-switched IgG1 in response to immunisation with T cell-dependent antigen [52], suggesting the *RHOH*-mapped variants may affect serum antibody levels through an impact on the efficiency of class switch recombination. A combination of shared signals, some with the same effect direction (immunoglobulin production) and others with the opposite (class switching efficiency), could explain the lack of overall genetic correlation between these isotypes.

The colocalising associations with concordant direction of effect included a novel association with IgA (lead SNP rs2872516), which colocalised with a known association of the same locus with IgM (rs4795397, PPC = 0.95). However, another novel association with IgG (rs17676923) in this region appeared to relate to a distinct causal variant with a posterior probability of distinct causal variants of ~1. This was supported by an examination of LD in the region, which showed that the lead SNPs for IgA and IgG were not in LD (r^2^ = 0.03), although those for IgA and IgM were (r^2^ = 0.83). All three SNPs belong to eQTL credible sets for *GSDMA*, *GSDMB* and *IKZF3* in blood.

**Fig. 2:**
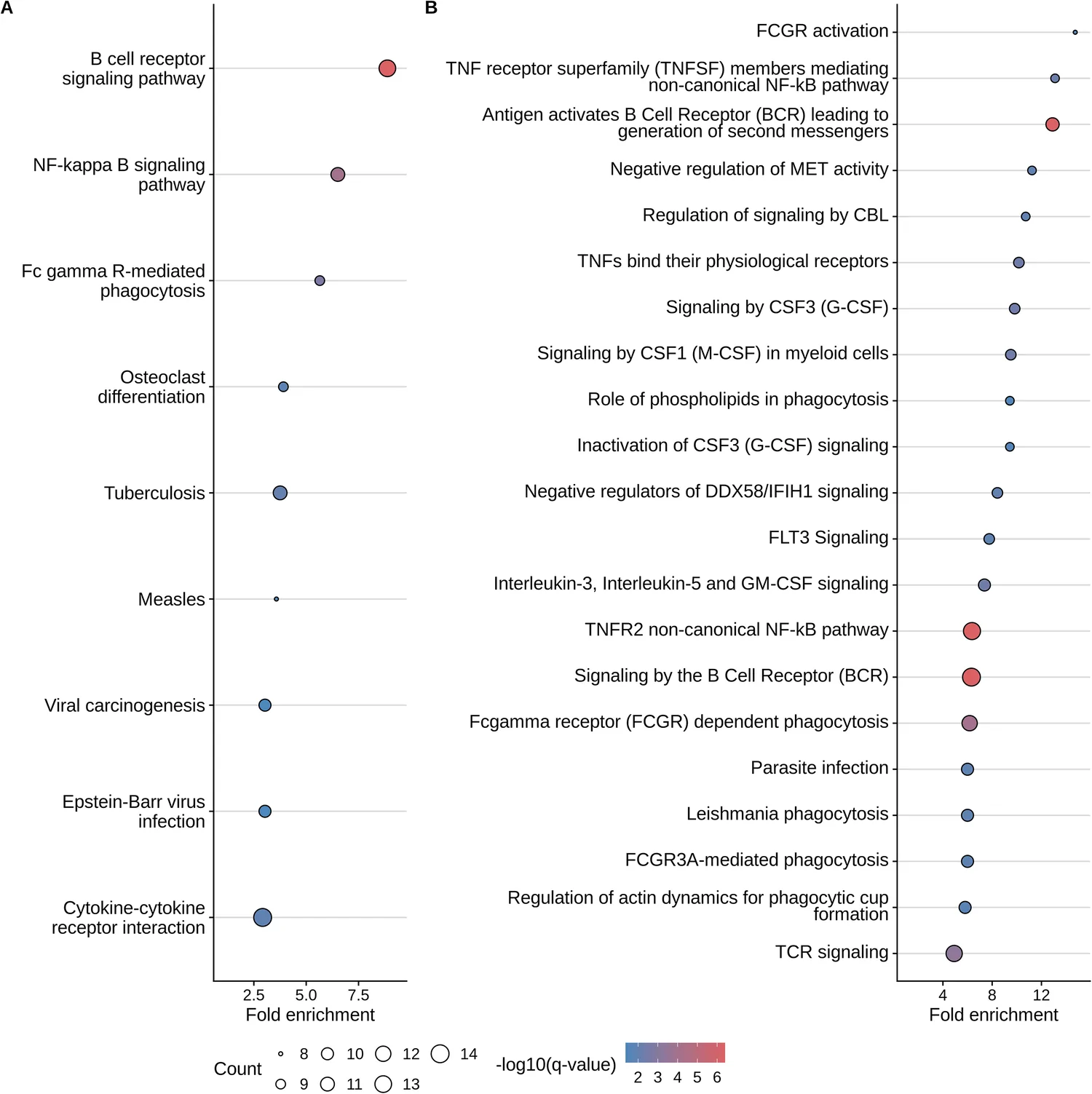
Significant pathways identified by the pathway analysis. **A** KEGG pathway analysis. **B** Reactome pathway analysis. The ‘fold enrichment’ is given by the ‘ratio of the frequency of input genes annotated in a term to the frequency of all genes annotated to that term’ [92].

Given the overlap of association signals, we used pathway analysis to summarise the associations seen across all isotypes. This identified six and ten significant KEGG and Reactome pathways, respectively (*q*-value < 0.05, Fig. 2), the most prominent amongst them relating to B cell receptor and NF-kB signaling. Our gene selection strategy was deliberately broad, which sometimes leads to the nomination of multiple genes for a single association. By comparing this list of genes with the list of genes in associated pathways we can highlight more likely causal genes in some cases (Supplementary Fig. 17). Of note, we see that the associated pathways typically contain associations with more than one isotype, highlighting the convergent biology of the GWAS associations in spite of the limited evidence of shared signals.

In a few cases, we were able to relate the genes of isotype-specific associations to isotype-specific biology. We mapped the IgM-associated SNP rs614004 to *CMTM7*, which encodes a membrane protein important in the development of innate-like B1 cells, which principally secrete constitutive, ‘natural’ IgM [53]. We mapped associations for both IgA and IgG (i.e. class-switched isotypes), but not for IgM, to *TRAF3*, which encodes a regulator of class switch recombination [54]. We linked the IgA-specific association on chromosome X to *C1GALT1C1*, essential for the O-glycosylation of IgA1 (the predominant IgA subclass in serum) and previously identified in a GWAS of galactose-deficient IgA1 (Gd-IgA1), an important IgA nephropathy endophenotype [55]. *ST3GAL1*, another IgA-specific gene, encodes an enzyme also participating in O-glycosylation of IgA1. A recent GWAS of Gd-IgA1 also identified *DERL3*, a gene in which we report a missense variant associated with serum IgA and which participates in the processing of misfolded proteins [56]. Despite studying a serum IgA phenotype, we also mapped an IgA-specific association to *LTBR*, essential for the development of Peyer’s patches, the site of secretory IgA production in the small intestine [57].

As antibodies are secreted by plasma cells and plasmablasts are derived from B cells, we hypothesised that some signals may relate to B cell abundance. Whilst we could not find any sufficiently large GWAS of B cell count for use in colocalisation, total lymphocyte count has been studied in large cohorts and we used one such GWAS as a proxy [27]. We found a modest but significant genetic correlation between serum IgA and lymphocyte count (r_g_ = 0.09, *p* = 3 × 10^–4^). Whilst r_g_ estimates for IgM and IgG were not significant, we conducted colocalisation analyses for all three isotypes with lymphocyte counts as r_g_ had not proved to be a dispositive indicator of cross-isotype colocalisation.

We found 16 loci with moderate to strong evidence of a shared causal variant for serum antibody and lymphocyte count phenotypes (Supplementary Table 12): nine for IgA, six for IgM and one for IgG (minimum PPC = 0.69), including the above-mentioned signal near *GSDMB*, which colocalised with both IgA and IgM. However, the directions of effect were not consistent: higher immunoglobulin levels were associated with higher lymphocyte counts in eight cases and with lower lymphocyte counts in the other eight. This explains the lack of detectable genetic correlation, which is a genome-wide measure. Our interpretation of the ostensibly paradoxical relationship in the latter eight cases is hampered by the lack of B cell-specific data. Given lymphocyte count is the sum of B, T and natural killer cell counts, an increased total lymphocyte count could arise from the production of more B cells or fewer in the context of more T cells or a change in T- or natural killer cell count absent any change in B-cell count.

All isotypes showed significant genetic correlation with two or more immune-mediated diseases (IMDs), amounting to nine significant estimates (Supplementary Table 13). These were positive with the exception of a negative genetic correlation between IgG and asthma (r_g_ = –0.1). We observed the strongest correlation between serum IgA and IgA nephropathy (r_g_ = 0.33), replicating published work [6]. Inclusion of the *IGH*, *IGK* and *IGL* loci made no nominally significant difference to antibody-immune disease genetic correlation estimates as assessed by z-test.

Several loci well-known for their association with IMDs colocalised with serum antibody signals, bolstering the evidence for the biological salience of the associations identified in our meta-analyses (Supplementary Fig. 18). IgA association signals at the *IL7R*, *INAVA*, *UBE2L3* and *IL2RA* loci colocalised with three to five IMDs each (minimum PPC = 0.72) and, with one exception, SNP effects were positively correlated between the serum IgA and IMD phenotypes. We observed antibody-IMD signal colocalisation at the aforementioned *GSDMB* locus for eight isotype-IMD pairs (minimum PPC = 0.76), including all three studied isotypes. The IgM association signal centred upon the *PTPN22* missense variant rs2476601 colocalised strongly with those of rheumatoid arthritis, type 1 diabetes, Crohn’s disease, systemic lupus erythematosus and hypothyroidism (minimum PPC = 1.0), IMDs whose association with this mutation is well-known [20, 24, 58]. In general we observed that the direction of effect for serum antibody- and IMD-associated SNPs was neither consistently concordant nor discordant (Supplementary Fig. 18). IgA nephropathy was exceptional in this regard: all four of its signals colocalising with serum IgA (minimum PPC = 0.86) showed positively correlated effects across the locus and at the lead SNP. Two of these signals mapped to the genes *TNFSF13* and *TNFRSF13B*, the APRIL-TACI receptor-ligand pair and a third mapped to another TNF family member, *TNFSF8*; all three genes are implicated in B-cell biology.

68 of our associations were in or near known genes associated with inborn errors of immunity (IEIs); 32 of these associations were novel (Supplementary Table 14). With the exception of the more common antibody deficiencies, IEI phenotypes do not admit formal colocalisation analyses for want of summary statistics and evidence of common-variant architecture. For IgM, these associations included variants in *CARD11*, *LYN* and *CIITA* as well as rs12554596, an intronic variant in and eQTL for *PAX5*, which encodes a transcription factor and ‘master regulator’ of B cell development [59]. A single case of PAX5 deficiency is known, featuring B-cell lymphopaenia and hypogammaglobulinaemia [60]. In two cases, variants associated with IgA and IgM appeared to represent distinct causal variants (on the basis of low LD and *coloc* favouring the distinct causal variant hypothesis), but both variants were linked to the same gene. IgM-associated rs290243 and IgA-associated rs4744011 are not in LD (r^2^ = 0.18), yet both belong to eQTL credible sets for *SYK* in NK cells and monocytes, respectively. *SYK* encodes a non-receptor tyrosine kinase which participates in BCR signalling [61] and in which gain-of-function mutations cause an autoinflammatory disorder [62]. Similarly, rs6589225 and rs4938518 were associated with IgM and IgA respectively. Again, we observed these to be in low LD (r^2^ = 0.28) whilst belonging to eQTL credible sets for the same gene, *POU2AF1*. A loss-of-function mutation in *POU2AF1* has been observed to cause an autosomal recessive, CVID-like antibody deficiency, BOB1 deficiency, in one patient [63].

Other notable associations include IgA with rs1585213, an intronic variant in *NFKB1*, which encodes NF-κB, a critical immune regulator downstream of TLR and B-cell receptor (BCR) signalling and integral to development of the humoral response [64]. Loss-of-function mutations in *NFKB1* have been identified as the most common cause of common variable immunodeficiency [65], an antibody deficiency characterised by IgG deficiency alongside IgA and/or IgM deficiency; IgA is absent in a majority of patients [66]. Similarly, rs6890853 belongs to e-, s- and pQTL credible sets for *IL7R*/*IL7Rɑ*, the expression and signalling of which are important in the early stage of B-cell progenitor development. Loss-of-function mutations in *IL7R* cause the B cell-positive severe combined immunodeficiency IL7R deficiency [67].

Just as our pathway analyses linked association-related genes to B-cell biology, we could relate many individual novel antibody associations to the same. We found three novel IgA associations featuring missense variants in B cell-related genes. rs13388394 is located in *RASGRP3*, which encodes a guanine nucleotide exchange factor and RAS activator. Its murine orthologue *Rasgrp3* contributes to B-cell receptor-induced Ras activation and the deletion of *Rasgrp3* leads to deficient production of certain IgG subclasses [68]. rs113495277 is a missense variant in *TRAF1*. TNF receptor associated factor 1 (TRAF1) forms multimeric complexes with TRAF2 which bind to the cytoplasmic domain of CD40 (the cell-surface receptor for the costimulatory ligand CD40L) upon ligation and activate the canonical NF-κB signalling pathway [69, 70].

rs3177243 is a missense variant in *DERL3*, a gene recently associated with B cell activation and differentiation and the unfolded protein response [71]. Its association with the latter may explain its expression in plasma cells given their burden of secretory protein synthesis; rs3177243 is also associated with an IgG N-glycan phenotype [72] and non-albumin serum protein levels [73]. We also identified a novel IgM-associated missense variant, rs11666267, in *NIBAN3*, which encodes B cell novel protein 1, a regulator of BCR signalling [74].

Other variants were linked to genes involved in B cell-related malignancies. This included two IgA-associated variants, rs55901664 and rs17505102, which were intronic to the genes *TBL1XR1* and *TP63*, respectively. Somatic mutations in *TBL1XR1* cause impaired plasma cell differentiation and a bias towards the production of immature memory B cells in aggressive B cell lymphomas [75], while *TP63* is known to participate in somatic gene fusion events with *TBL1XR1* in a small minority of diffuse large B-cell lymphomas [76]. The IgG-associated variant rs184552867 is an intronic variant in *PRDM11* which has been identified as a tumour suppressor gene in B cells and which may be downregulated in diffuse large B-cell lymphoma [77].

## Discussion

The *IGH* and *IGK* loci encode immunoglobulin chains and are expected to be of central importance in a genetic analysis of serum antibody production. Indeed, germline variation within these loci has known effects upon the antibody repertoire and disease liability [78–81]. However, the large and (within isotypes) discordant directions of effect we observed for serum IgG and serum IgA at the *IGH* locus suggest an artefactual epitope effect, particularly for the missense variant rs117775520.

As we cannot confidently report estimates obtained in these loci, we believe that the heritability estimates (and genetic correlation estimates derived from them) reported without the inclusion of the *IGH*, *IGK* and *IGL* loci and without the MHC, are more reliable. These estimates are also valuable in their own right as measures of the partial heritability of these traits attributable to genetic variation outside these important regions. We note that SumHer’s heritability estimation procedure depends upon a random effects model unsuited to isolated large effect sizes and the extreme LD seen in these regions. We saw an appreciable increase in estimated heritability with inclusion of the MHC, albeit calculated naively. Using long-read sequencing to improve resolution in the *IGH*, *IGK* and *IGL* regions [82] together with more sophisticated analysis will be required for accurate determination of the heritability they explain in serum antibody levels. Structural variation in these regions, the study of which is made possible by long-read sequencing, has already been shown to affect the antibody repertoire and isotype abundance; this variation includes copy number variation in *IGHA1* [83, 84]. As we mapped strong signals to immunoglobulin heavy chain genes encoding distinct IgA and IgG subclasses (rs117775520 in particular is a missense variant in *IGHA1*), subclass-specific GWAS combined with long-read sequencing might provide the multimodal increase in resolution necessary to better understand these associations.

We detected positive phenotypic and genetic correlations between antibody isotypes but limited overlap in their individual association signals. This suggests that our cross-isotype genetic correlation estimates are driven by shared variant effects which do not achieve genome-wide significance despite our large sample sizes. Our list of associations thus remains an incomplete catalogue of the common-variant determinants of serum antibody production; we also report a SNP heritability estimate for serum IgA (0.16) which falls far short of the heritability estimate obtained from a past twin study (0.50) and the SNP heritability estimate (0.31) obtained in the same twin cohort [5]. The effects we did detect reflect both known and novel biological mechanisms impacting immunoglobulin secretion, including B-cell progenitor development, antibody production and class switching and the role of helper T cells in stimulating immunoglobulin production. Despite comparably-sized samples for IgG and IgM, we detected far fewer associations for the former and estimated its heritability to be around one third of the latter’s. Outside of the *IGH*, *IGK* and *IGL* loci, we do not believe technical effects can account for the lack of cross-isotype colocalisation. Each of our meta-analyses incorporated samples both from GWAS using immunoassays and those using aptamer-based assays. The composition with respect to assay type was similar for IgG and IgM: 79% and 84% came from aptamer-based studies for IgG and IgM, respectively and 14% from immunoassay-based studies for both. The proportion of samples taken from immunoassay-based studies (35%) was higher for IgA and that taken from aptamer-based studies (54%) lower, because of our inclusion of the immunoassay-based study by Liu et al. We believe it is unlikely that differences in sample composition between isotypes could introduce locus-specific biases in colocalisation outside of the immunoglobulin chain loci and in the face of the evidence of average genome-wide shared signal our genetic correlation estimates represent. More plausible is the possibility that the aggregation of IgG subclasses obscures subclass-specific colocalisations with IgM and IgA. Given recent evidence of IgG subclass-specific genetic architecture [80], a possible explanation for the smaller number of IgG associations we report is that the larger number of IgG subclasses (four compared with IgA’s two and IgM’s lack thereof) and our aggregation of them into one phenotype may have acted to dilute subclass-specific association signals. Future work could extend IgG and IgA subclass-specific GWAS to the same large scale as the meta-analyses we present here to clarify the subclasses driving the associations we report.

We found evidence of common-variant architecture shared between antibody phenotypes and immune-related traits. For the latter we observed multiple colocalisation signals indicative of shared causal variants in the absence of strong genetic correlation between lymphocyte count and the antibody phenotypes. Our expectation of straightforward concordance between antibody and lymphocyte count effect directions was refuted by the lack of a clear relationship. We observed the same inconsistency in effect direction across antibody signals colocalising with immune-mediated disease signals. These results suggest that immunoglobulin levels are not themselves on the causal path for any of these diseases, with the probable exception of IgA nephropathy [6]. The shared associations instead reflect processes that lead to change in both immune-mediated disease risk and immunoglobulin levels. Our results also provide a cautionary tale for the use of genetic correlation more generally: weak or absent genetic correlation, as with lymphocyte counts, should not be taken as a dispositive indicator of genetic independence and positive genetic correlation may be consistent with the presence of shared causal variants which nonetheless exert discordant effects on each phenotype.

We have presented an expanded view of the genetic architecture of serum antibody production. This view will be broadened still further by the adoption of modern high-throughput affinity-based proteomics techniques, such as the ‘SomaScan’ DNA aptamer-based method used to generate the Pietzner and Eldjarn datasets included in our meta-analyses [85]. These methods allow many proteins to be assayed in parallel and should make the measurement of serum proteins in large, biobank-sized cohorts affordable [86]. Investigation of the pattern of shared genetic effects on lymphocyte abundance and antibody production and the seemingly paradoxical disagreement in the direction of variant effects upon them, was hindered by the lack of large, publicly available B and T cell-specific phenotype GWAS. Should such datasets become accessible, their use in colocalisation analysis should prove insightful as to the extent to which variants mediate their effect on serum antibody levels through proximal effects on lymphocyte abundance. The composite nature of the lymphocyte count phenotype in the available dataset prevented investigation of the causal effect of the abundance of B cells (or of relevant T helper cell subsets) on serum antibody production through Mendelian randomisation. B-cell lymphopaenia underlies hypo- and agammaglobulinaemia in a number of antibody deficiencies, such as Bruton’s agammaglobulinaemia and some CVID-like disorders such as TRNT1 deficiency [35]. This suggests smaller genetic effects on B cell abundance mediated by common variants might also underlie variation in serum antibody production in the normophysiological setting.

We meta-analysed GWAS conducted almost exclusively in individuals of European ancestry, the sole exception being the trans-ancestry GWAS of serum IgA by Liu et al. We were thus not able to investigate the extent to which the genetic architecture of serum antibody production varies across ancestries or the replicability of our discoveries across populations. Whilst GWAS of antibody phenotypes in East Asian ancestries have been published [8, 87], their summary statistics are not publicly available for meta-analysis, a common obstacle in GWAS meta-analysis [88].

As well as IMDs generally, our results are relevant to the study of the antibody deficiencies, particularly the two most common, selective IgA deficiency and common variable immunodeficiency [3]. The genetic aetiology of these diseases remains obscure in most patients and there is evidence of a common-variant contribution to both [10, 89, 90]. They are therefore good candidates for joint genetic analysis with the meta-analyses we publish here. Such an analysis could address the question of whether inheritance of a preponderance of common hypomorphic serum antibody-associated variants could account for clinical antibody deficiency in cases where a Mendelian pattern of disease or known monogenic causal variant is absent.

## Supplementary information

Supplementary Figures and Supplementary Table captions

Supplementary Tables

## Data Availability

GWAS summary statistics have been deposited at the following Zenodo URL: https://doi.org/10.5281/zenodo.17010403.

## Code availability

We have made available the code used to produce the results presented here in the form of a *snakemake* [91] pipeline (https://doi.org/10.5281/zenodo.18023612): https://github.com/twillis209/serum_ig_pipeline. The standalone *snakemake* pipeline used to process 1kGP data is also available: https://github.com/twillis209/1kGP_pipeline.

## References

[1] Hatagima A, Cabello PH, Krieger H. Causal analysis of the variability of IgA, IgG, and IgM immunoglobulin levels. Hum Biol. 1999;71:219–29. 10222644

[2] Bruton OC. AGAMMAGLOBULINEMIA. Pediatrics. 1952;9:722–8. 14929630

[3] Pan-Hammarström Q, Hammarström L. Antibody deficiency diseases. Eur J Immunol. 2008;38:327–33. 10.1002/eji.200737927 18200502

[4] Yazdani R, Fekrvand S, Shahkarami S, Azizi G, Moazzami B, Abolhassani H, et al. The hyper IgM syndromes: epidemiology, pathogenesis, clinical manifestations, diagnosis and management. Clin Immunol. 2019;198:19–30. 10.1016/j.clim.2018.11.007 30439505

[5] Viktorin A, Frankowiack M, Padyukov L, Chang Z, Melén E, Sääf A, et al. IgA measurements in over 12,000 Swedish twins reveal sex differential heritability and a regulatory locus near CD30L. Hum Mol Genet. 2014;23:4177–84. 10.1093/hmg/ddu135 PMC4082371 24676358

[6] Liu L, Khan A, Sanchez-Rodriguez E, Zanoni F, Li Y, Steers N, et al. Genetic regulation of serum IgA levels and susceptibility to common immune, infectious, kidney, and cardio-metabolic traits. Nat Commun. 2022;13:6859. 10.1038/s41467-022-34456-6 PMC9651905 36369178

[7] Bian S, Guo X, Yang X, Wei Y, Yang Z, Cheng S, et al. Genetic determinants of IgG antibody response to COVID-19 vaccination. Am J Hum Genet. 2024;111:181–99. 10.1016/j.ajhg.2023.12.005 PMC10806743 38181733

[8] Yang M, Wu Y, Lu Y, Liu C, Sun J, Liao M, et al. Genome-wide scan identifies variant in TNFSF13 associated with serum IgM in a healthy Chinese male population. PLoS One. 2012;7:e47990. 10.1371/journal.pone.0047990 PMC3485370 23118916

[9] Kim J-H, Cheong HS, Park JS, Jang A-S, Uh S-T, Kim Y-H, et al. A genome-wide association study of total serum and mite-specific IgEs in asthma patients. PLoS One. 2013;8:e71958. 10.1371/journal.pone.0071958 PMC3742455 23967269

[10] Willis TW, Gkrania-Klotsas E, Wareham NJ, McKinney EF, Lyons PA, Smith KGC, et al. Leveraging pleiotropy identifies common-variant associations with selective IgA deficiency. Clin Immunol. 2024;268:110356. 10.1016/j.clim.2024.110356 PMC7618579 39241920

[11] Dennis JK, Sealock JM, Straub P, Lee YH, Hucks D, Actkins K, et al. Clinical laboratory test-wide association scan of polygenic scores identifies biomarkers of complex disease. Genome Med. 2021;13:6. 10.1186/s13073-020-00820-8 PMC7807864 33441150

[12] Scepanovic P, Alanio C, Hammer C, Hodel F, Bergstedt J, Patin E, et al. Human genetic variants and age are the strongest predictors of humoral immune responses to common pathogens and vaccines. Genome Med. 2018;10:59. 10.1186/s13073-018-0568-8 PMC6063007 30053915

[13] Eldjarn GH, Ferkingstad E, Lund SH, Helgason H, Magnusson OT, Gunnarsdottir K, et al. Large-scale plasma proteomics comparisons through genetics and disease associations. Nature. 2023;622:348–58. 10.1038/s41586-023-06563-x PMC10567571 37794188

[14] Pietzner M, Wheeler E, Carrasco-Zanini J, Cortes A, Koprulu M, Wörheide MA, et al. Mapping the proteo-genomic convergence of human diseases. Science. 2021;374:eabj1541. 10.1126/science.abj1541 PMC9904207 34648354

[15] Jonsson S, Sveinbjornsson G, de Lapuente Portilla AL, Swaminathan B, Plomp R, Dekkers G, et al. Identification of sequence variants influencing immunoglobulin levels. Nat Genet. 2017;49:1182–91. 10.1038/ng.3897 28628107

[16] Cordell HJ, Fryett JJ, Ueno K, Darlay R, Aiba Y, Hitomi Y, et al. An International Genome-Wide Meta-Analysis of Primary Biliary Cholangitis: Novel Risk Loci and Candidate Drugs. J Hepatol. 2021;75:572–81. 10.1016/j.jhep.2021.04.055 PMC8811537 34033851

[17] Ji S-G, Juran BD, Mucha S, Folseraas T, Jostins L, Melum E, et al. Genome-wide association study of primary sclerosing cholangitis identifies new risk loci and quantifies the genetic relationship with inflammatory bowel disease. Nat Genet. 2017;49:269–73. 10.1038/ng.3745 PMC5540332 27992413

[18] Ishigaki K, Sakaue S, Terao C, Luo Y, Sonehara K, Yamaguchi K, et al. Multi-ancestry genome-wide association analyses identify novel genetic mechanisms in rheumatoid arthritis. Nat Genet. 2022;54:1640–51. 10.1038/s41588-022-01213-w PMC10165422 36333501

[19] Bentham J, Morris DL, Cunninghame Graham DS, Pinder CL, Tombleson P, Behrens TW, et al. Genetic association analyses implicate aberrant regulation of innate and adaptive immunity genes in the pathogenesis of systemic lupus erythematosus. Nat Genet. 2015;47:1457–64. 10.1038/ng.3434 PMC4668589 26502338

[20] de Lange KM, Moutsianas L, Lee JC, Lamb CA, Luo Y, Kennedy NA, et al. Genome-wide association study implicates immune activation of multiple integrin genes in inflammatory bowel disease. Nat Genet. 2017;49:256–61. 10.1038/ng.3760 PMC5289481 28067908

[21] Chiou J, Geusz RJ, Okino M-L, Han JY, Miller M, Melton R, et al. Interpreting type 1 diabetes risk with genetics and single-cell epigenomics. Nature. 2021;594:398–402. 10.1038/s41586-021-03552-w PMC10560508 34012112

[22] International Multiple Sclerosis Genetics Consortium. Multiple sclerosis genomic map implicates peripheral immune cells and microglia in susceptibility. Science. 2019;365:eaav7188. 10.1126/science.aav7188 PMC7241648 31604244

[23] Pasanen A, Sliz E, Huilaja L, Reimann E, Mägi R, et al. FinnGen; Estonian Biobank Research Team Identifying atopic dermatitis risk loci in 1,094,060 individuals with subanalysis of disease severity and onset. J Investig Dermatol. 2024;144:2417–25. 10.1016/j.jid.2024.02.036 38663478

[24] Figuerêdo J, Krebs K, Pujol-Gualdo N, Haller T, Võsa U, Volke V, et al. Uncovering the shared genetic components of thyroid disorders and reproductive health. Eur J Endocrinol. 2024;191:211–22. 10.1093/ejendo/lvae094 39067062

[25] Trynka G, Hunt KA, Bockett NA, Romanos J, Mistry V, Szperl A, et al. Dense genotyping identifies and localizes multiple common and rare variant association signals in celiac disease. Nat Genet. 2011;43:1193–201. 10.1038/ng.998 PMC3242065 22057235

[26] Kiryluk K, Sanchez-Rodriguez E, Zhou X-J, Zanoni F, Liu L, Mladkova N, et al. Genome-wide association analyses define pathogenic signaling pathways and prioritize drug targets for IgA nephropathy. Nat Genet. 2023;55:1091–105. 10.1038/s41588-023-01422-x PMC11824687 37337107

[27] Chen M-H, Raffield LM, Mousas A, Sakaue S, Huffman JE, Moscati A, et al. Trans-ethnic and ancestry-specific blood-cell genetics in 746,667 individuals from 5 global populations. Cell. 2020;182:1198–1213.e14. 10.1016/j.cell.2020.06.045 PMC7480402 32888493

[28] Cerezo M, Sollis E, Ji Y, Lewis E, Abid A, Bircan KO, et al. The NHGRI-EBI GWAS Catalog: standards for reusability, sustainability and diversity. Nucleic Acids Res. 2025;53:D998–1005. 10.1093/nar/gkae1070 PMC11701593 39530240

[29] Chang CC, Chow CC, Tellier LC, Vattikuti S, Purcell SM, Lee JJ. Second-Generation PLINK: rising to the challenge of larger and richer datasets. Gigascience. 2015;4:s13742-015–0047–8. 10.1186/s13742-015-0047-8 PMC4342193 25722852

[30] Purcell S, Chang C PLINK 2.0. https://www.cog-genomics.org/plink/2.0/general_usage#cite.

[31] Singh A, Southam L, Hatzikotoulas K, Rayner NW, Suzuki K, Taylor HJ, et al. Correcting for genomic inflation leads to a loss of power in large-scale genome-wide association study meta-analysis. Genet Epidemiol. 2025;49:e70016. 10.1002/gepi.70016 PMC12327166 40767503

[32] Lewis MJ, Wang S. locuszoomr: an R package for visualizing publication-ready regional gene locus plots. Bioinform Adv. 2024;5:vbaf006. 10.1093/bioadv/vbaf006 PMC11912002 40099154

[33] Machiela MJ, Chanock SJ. LDlink: a web-based application for exploring population-specific haplotype structure and linking correlated alleles of possible functional variants. Bioinformatics. 2015;31:3555–7. 10.1093/bioinformatics/btv402 PMC4626747 26139635

[34] Gudjonsson A, Gudmundsdottir V, Axelsson GT, Gudmundsson EF, Jonsson BG, Launer LJ, et al. A genome-wide association study of serum proteins reveals shared loci with common diseases. Nat Commun. 2022;13:480. 10.1038/s41467-021-27850-z PMC8789779 35078996

[35] Poli MC, Aksentijevich I, Bousfiha AA, Cunningham-Rundles C, Hambleton S, Klein C, et al. Human inborn errors of immunity: 2024 update on the classification from the International Union of Immunological Societies Expert Committee. J Human Immun. 2025. 10.70962/jhi.20250003. PMC12829761 41608114

[36] Durinck S, Spellman PT, Birney E, Huber W. Mapping identifiers for the integration of genomic datasets with the R/Bioconductor package biomaRt. Nat Protoc. 2009;4:1184–91. 10.1038/nprot.2009.97 PMC3159387 19617889

[37] Durinck S, Moreau Y, Kasprzyk A, Davis S, De Moor B, Brazma A, et al. BioMart and Bioconductor: a powerful link between biological databases and microarray data analysis. Bioinformatics. 2005;21:3439–40. 10.1093/bioinformatics/bti525 16082012

[38] Speed D, Balding DJ. SumHer better estimates the SNP heritability of complex traits from summary statistics. Nat Genet. 2019;51:277–84. 10.1038/s41588-018-0279-5 PMC6485398 30510236

[39] A global reference for human genetic variation. Nature. 2015;526:68–74. 10.1038/nature15393 PMC4750478 26432245

[40] Byrska-Bishop M, Evani US, Zhao X, Basile AO, Abel HJ, Regier AA, et al. High-coverage whole-genome sequencing of the expanded 1000 Genomes Project cohort, including 602 trios. Cell. 2022;185:3426–3440.e19. 10.1016/j.cell.2022.08.004 PMC9439720 36055201

[41] Buniello A, Suveges D, Cruz-Castillo C, Llinares MB, Cornu H, Lopez I, et al. Open targets platform: facilitating therapeutic hypotheses building in drug discovery. Nucleic Acids Res. 2025;53:D1467–75. 10.1093/nar/gkae1128 PMC11701534 39657122

[42] Dyer SC, Austine-Orimoloye O, Azov AG, Barba M, Barnes I, Barrera-Enriquez VP, et al. Ensembl 2025. Nucleic Acids Res. 2025;53:D948–57. 10.1093/nar/gkae1071 PMC11701638 39656687

[43] Yu G. Thirteen years of clusterProfiler. Innovations. 2024;5:100722. 10.1016/j.xinn.2024.100722 PMC11551487 39529960

[44] Yu G, He Q-Y. ReactomePA: an R/Bioconductor package for reactome pathway analysis and visualization. Mol Biosyst. 2016;12:477–9. 10.1039/c5mb00663e 26661513

[45] Giambartolomei C, Vukcevic D, Schadt EE, Franke L, Hingorani AD, Wallace C, et al. Bayesian test for colocalisation between pairs of genetic association studies using summary statistics. PLoS Genet. 2014;10:e1004383. 10.1371/journal.pgen.1004383 PMC4022491 24830394

[46] Wakefield J. Bayes factors for genome-wide association studies: comparison with *P*-values. Genet Epidemiol. 2009;33:79–86. 10.1002/gepi.20359 18642345

[47] Kanai M, Elzur R, Zhou W, Day MJ, Finucane HKGlobal biobank meta-analysis initiative Meta-analysis fine-mapping is often miscalibrated at single-variant resolution. Cell Genom. 2022;2:100210. 10.1016/j.xgen.2022.100210 PMC9839193 36643910

[48] Jefferis R, Lefranc M-P. Human immunoglobulin allotypes: possible implications for immunogenicity. MAbs. 2009;1:332–8. 10.4161/mabs.1.4.9122 PMC2726606 20073133

[49] Purcell RA, Aurelia LC, Esterbauer R, Allen LF, Bond KA, Williamson DA, et al. Immunoglobulin G genetic variation can confound assessment of antibody levels via altered binding to detection reagents. Clin Transl. Immunology. 2024;13:e1494. 10.1002/cti2.1494 PMC10902689 38433763

[50] Li X, Bu X, Lu B, Avraham H, Flavell RA, Lim B. The hematopoiesis-specific GTP-binding protein RhoH is GTPase-deficient and modulates the activities of other Rho GTPases by an inhibitory function. Mol Cell Biol. 2002;22:1158–71. 10.1128/MCB.22.4.1158-1171.2002 PMC134637 11809807

[51] Crequer A, Troeger A, Patin E, Ma CS, Picard C, Pedergnana V, et al. Human RHOH deficiency causes T cell defects and susceptibility to EV-HPV infections. J Clin Invest. 2012;122:3239–47. 10.1172/JCI62949 PMC3428089 22850876

[52] Dorn T. Analysis of RhoH function in vivo. [Ludwig-Maximilians-Universität München]: Ludwig-Maximilians-Universität München; 2007. Available from: https://edoc.ub.uni-muenchen.de/7457/1/Dorn_Tatjana.pdf.

[53] Liu Z, Liu Y, Li T, Wang P, Mo X, Lv P, et al. CMTM7 plays key roles in TLR-induced plasma cell differentiation and p38 activation in murine B-1 B cells. Eur J Immunol. 2020;50:809–21. 10.1002/eji.201948363 32022930

[54] Chen Z, Krinsky A, Woolaver RA, Wang X, Chen SMY, Popolizio V, et al. TRAF3 acts as a checkpoint of B cell receptor signaling to control antibody class switch recombination and anergy. J Immunol. 2020;205:830–41. 10.4049/jimmunol.2000322 PMC7369235 32591397

[55] Kiryluk K, Li Y, Moldoveanu Z, Suzuki H, Reily C, Hou P, et al. GWAS for serum galactose-deficient IgA1 implicates critical genes of the O-glycosylation pathway. PLoS Genet. 2017;13:e1006609. 10.1371/journal.pgen.1006609 PMC5328405 28187132

[56] Liu L, Shen A, Taylor AD, Atlas Khan, Reily C, Hall SD, et al. Genome-wide association study (GWAS) of serum galactose-deficient IgA1 uncovers shared genetic determinants with IgAN: Th-po0633. J Am Soc Nephrol. 2025;36:10.1681/ASN.2025pyp4sfbe.

[57] Fütterer A, Mink K, Luz A, Kosco-Vilbois MH, Pfeffer K. The lymphotoxin β receptor controls organogenesis and affinity maturation in peripheral lymphoid tissues. Immunity. 1998;9:59–70. 10.1016/s1074-7613(00)80588-9 9697836

[58] Eyre S, Hinks A, Bowes J, Flynn E, Martin P, Wilson AG, et al. Overlapping genetic susceptibility variants between three autoimmune disorders: rheumatoid arthritis, type 1 diabetes and coeliac disease. Arthritis Res Ther. 2010;12:R175. 10.1186/ar3139 PMC2991006 20854658

[59] Medvedovic J, Ebert A, Tagoh H, Busslinger M. Pax5: a master regulator of B cell development and leukemogenesis. Adv Immunol. 2011;111:179–206. 10.1016/B978-0-12-385991-4.00005-2 21970955

[60] Kaiser FMP, Gruenbacher S, Oyaga MR, Nio E, Jaritz M, Sun Q, et al. Biallelic PAX5 mutations cause hypogammaglobulinemia, sensorimotor deficits, and autism spectrum disorder. J Exp Med. 2025;219. Available from: https://rupress.org/jem/article-pdf/219/9/e20220498/1916562/jem_20220498.pdf. 10.1084/jem.20220498 PMC9372349 35947077

[61] Ackermann JA, Nys J, Schweighoffer E, McCleary S, Smithers N, Tybulewicz VLJ. Syk tyrosine kinase is critical for B cell antibody responses and memory B cell survival. J Immunol. 2015;194:4650–6. 10.4049/jimmunol.1500461 PMC4416743 25862820

[62] Wang L, Aschenbrenner D, Zeng Z, Cao X, Mayr D, Mehta M, et al. Gain-of-function variants in SYK cause immune dysregulation and systemic inflammation in humans and mice. Nat Genet. 2021;53:500–10. 10.1038/s41588-021-00803-4 PMC8245161 33782605

[63] Kury P, Staniek J, Wegehaupt O, Janowska I, Eckenweiler M, Korinthenberg R, et al. Agammaglobulinemia with normal B-cell numbers in a patient lacking Bob1. J Allergy Clin Immunol. 2021y;147:1977–80. 10.1016/j.jaci.2021.01.027 33571536

[64] Guldenpfennig C, Teixeiro E, Daniels M. NF-kB’s contribution to B cell fate decisions. Front Immunol. 2023;14:1214095. 10.3389/fimmu.2023.1214095 PMC10391175 37533858

[65] Tuijnenburg P, Lango Allen H, Burns SO, Greene D, Jansen MH, Staples E, et al. Loss-of-function nuclear factor κB subunit 1 (NFKB1) variants are the most common monogenic cause of common variable immunodeficiency in Europeans. J Allergy Clin Immunol. 2018;142:1285–96. 10.1016/j.jaci.2018.01.039 PMC6148345 29477724

[66] Bonilla FA, Barlan I, Chapel H, Costa-Carvalho BT, Cunningham-Rundles C, de la Morena MT, et al. International consensus document (ICON): common variable immunodeficiency disorders. J Allergy Clin Immunol Pr. 2016;4:38–59. 10.1016/j.jaip.2015.07.025 PMC4869529 26563668

[67] Kaiser FMP, Janowska I, Menafra R, de Gier M, Korzhenevich J, Pico-Knijnenburg I, et al. IL-7 receptor signaling drives human B-cell progenitor differentiation and expansion. Blood. 2023;142:1113–30. 10.1182/blood.2023019721 PMC10644098 37369082

[68] Coughlin JJ, Stang SL, Dower NA, Stone JC. RasGRP1 and RasGRP3 regulate B cell proliferation by facilitating B cell receptor-Ras signaling. J Immunol. 2005;175:7179–84. 10.4049/jimmunol.175.11.7179 16301621

[69] Elgueta R, Benson MJ, de Vries VC, Wasiuk A, Guo Y, Noelle RJ. Molecular mechanism and function of CD40/CD40L engagement in the immune system. Immunol Rev. 2009;229:152–72. 10.1111/j.1600-065X.2009.00782.x PMC3826168 19426221

[70] Fotin-Mleczek M, Henkler F, Hausser A, Glauner H, Samel D, Graness A, et al. Tumor necrosis factor receptor-associated factor (TRAF) 1 regulates CD40-induced TRAF2-mediated NF-kappaB activation. J Biol Chem. 2004;279:677–85. 10.1074/jbc.M310969200 14557256

[71] Lin L, Lin G, Lin H, Chen L, Chen X, Lin Q, et al. Integrated profiling of endoplasmic reticulum stress-related DERL3 in the prognostic and immune features of lung adenocarcinoma. Front Immunol. 2022;13:906420. 10.3389/fimmu.2022.906420 PMC9585215 36275646

[72] Landini A, Trbojević-Akmačić I, Navarro P, Tsepilov YA, Sharapov SZ, Vučković F, et al. Genetic regulation of post-translational modification of two distinct proteins. Nat Commun. 2022;13:1586. 10.1038/s41467-022-29189-5 PMC8948205 35332118

[73] Sinnott-Armstrong N, Tanigawa Y, Amar D, Mars N, Benner C, Aguirre M, et al. Genetics of 35 blood and urine biomarkers in the UK Biobank. Nat Genet. 2021;53:185–94. 10.1038/s41588-020-00757-z PMC7867639 33462484

[74] Hong R, Lai N, Ouchida R, Xiong E, Zhou Y, Min Q, et al. The B cell novel protein 1 (BCNP1) regulates BCR signaling and B cell apoptosis. Eur J Immunol. 2019;49:911–7. 10.1002/eji.201847985 30888050

[75] Venturutti L, Teater M, Zhai A, Chadburn A, Babiker L, Kim D, et al. TBL1XR1 mutations drive extranodal lymphoma by inducing a pro-tumorigenic memory fate. Cell. 2020;182:297–316.e27. 10.1016/j.cell.2020.05.049 PMC7384961 32619424

[76] Scott DW, Mungall KL, Ben-Neriah S, Rogic S, Morin RD, Slack GW, et al. TBL1XR1/TP63: a novel recurrent gene fusion in B-cell non-Hodgkin lymphoma. Blood. 2012;119:4949–52. 10.1182/blood-2012-02-414441 PMC3367896 22496164

[77] Asmar F, Fog CK, Jensen K, Jacobsen L, Ralfkiær E, Geisler CH, et al. PRDM11 is a novel putative tumor suppressor in aggressive B-cell lymphoma. Blood. 2010;116:1002–1002.

[78] Rodriguez OL, Safonova Y, Silver CA, Shields K, Gibson WS, Kos JT, et al. Genetic variation in the immunoglobulin heavy chain locus shapes the human antibody repertoire. Nat Commun. 2023;14:4419. 10.1038/s41467-023-40070-x PMC10362067 37479682

[79] Engelbrecht E, Rodriguez OL, Lees W, Vanwinkle Z, Shields K, Schultze S, et al. Germline polymorphisms in the immunoglobulin kappa and lambda loci underpinning antibody light chain repertoire variability. Nat Commun. 2025;16:11707. 10.1038/s41467-025-66759-9 PMC12753720 41315391

[80] Olafsdottir TA, Thorleifsson G, Lopez de Lapuente Portilla A, Jonsson S, Stefansdottir L, Niroula A, et al. Sequence variants influencing the regulation of serum IgG subclass levels. Nat Commun. 2024;15:8054. 10.1038/s41467-024-52470-8 PMC11401918 39277589

[81] Corcoran M, Narang S, Kaduk M, Chernyshev M, Färnert A, Sundling C, et al. Ultra-high-throughput IGH genotyping of 25 global populations reveals population-biased allelic diversity and homozygous V and D gene deletions. Immunity. 2026;59:1107–1122.e5. 10.1016/j.immuni.2026.01.026 41844150

[82] Watson CT, Collins AM, Ohlin M, Heather JM, Peres A, Lees WD, et al. Building immunoglobulin and T cell receptor gene databases for the future. Immunoinformatics. 2025;19:100059.

[83] Jana U, Rodriguez OL, Lees W, Engelbrecht E, Vanwinkle Z, Peres A, et al. The human IG heavy chain constant gene locus is enriched for large structural variants and coding polymorphisms that vary among human populations. Cell Genom. 2025;101058:101058. 10.1016/j.xgen.2025.101058 PMC12926203 41151584

[84] Gornitzka MB, Røsjø E, Jana U, Ford EE, Tourancheau A, Lees WD, et al. Ultra-long sequencing for contiguous haplotype resolution of the human immunoglobulin heavy-chain locus. Genome Res. 2025;35:2240–51. 10.1101/gr.280400.125 PMC12487824 40841171

[85] Gold L, Walker JJ, Wilcox SK, Williams S. Advances in human proteomics at high scale with the SOMAscan proteomics platform. N Biotechnol. 2012;29:543–9. 10.1016/j.nbt.2011.11.016 22155539

[86] Largest proteome study enlists 14 biopharmas. Nat Biotechnol. 2025;43:153. 10.1038/s41587-025-02577-0 39953225

[87] Kanai M, Akiyama M, Takahashi A, Matoba N, Momozawa Y, Ikeda M, et al. Genetic analysis of quantitative traits in the Japanese population links cell types to complex human diseases. Nat Genet. 2018;50:390–400. 10.1038/s41588-018-0047-6 29403010

[88] Reales G, Wallace C. Sharing GWAS Summary Statistics Results in More Citations. Communications. Biology. 2023;6:1–6. 10.1038/s42003-023-04497-8 PMC9884206 36709395

[89] Li J, Jørgensen SF, Maggadottir SM, Bakay M, Warnatz K, Glessner J, et al. Association of CLEC16A with human common variable immunodeficiency disorder and role in murine B cells. Nat Commun. 2015;6:6804. 10.1038/ncomms7804 PMC4444044 25891430

[90] Bronson PG, Chang D, Bhangale T, Seldin MF, Ferreira RC, Urcelay E, et al. Common variants at PVT1, ATG13-AMBRA1, AHI1 and CLEC16A are associated with selective IgA deficiency. Nat Genet. 2016;48:1425–9. 10.1038/ng.3675 PMC5086090 27723758

[91] Mölder F, Jablonski KP, Letcher B, Hall MB, Tomkins-Tinch CH, Sochat V, et al. Sustainable data analysis with snakemake. F1000Res. 2022;10. Available from: 10.12688/f1000research.29032.2. PMC8114187 34035898

[92] Wu T, Hu E, Xu S, Chen M, Guo P, Dai Z, et al. clusterProfiler 4.0: a universal enrichment tool for interpreting omics data. Innovations. 2021;2:100141. 10.1016/j.xinn.2021.100141 PMC8454663 34557778

